# Selective inhibition of nuclear export: a promising approach in the shifting treatment paradigms for hematological neoplasms

**DOI:** 10.1038/s41375-021-01483-z

**Published:** 2022-01-29

**Authors:** Suresh Kumar Balasubramanian, Asfar S. Azmi, Jaroslaw Maciejewski

**Affiliations:** 1 Department of Oncology, Wayne State University School of Medicine, Detroit, USA; 2 Translational Hematology and Oncology Research, Cleveland Clinic, Cleveland, USA

**Keywords:** Targeted therapies, Drug development

## Abstract

Novel targeted therapeutics alone or in rational combinations are likely to dominate the future management of various hematological neoplasms. However, the challenges currently faced are the molecular heterogeneity in driver lesions and genetic plasticity leading to multiple resistance pathways. Thus, progress has overall been gradual. For example, despite the advent of targeted agents against actionable drivers like *FLT3* in acute myeloid leukemia (AML), the prognosis remains suboptimal in newly diagnosed and dismal in the relapsed/refractory (R/R) setting, due to other molecular abnormalities contributing to inherent and acquired treatment resistance. Nuclear export inhibitors are of keen interest because they can inhibit several active tumorigenic processes simultaneously and also synergize with other targeted drugs and chemotherapy. XPO1 (or CRM1, chromosome maintenance region 1) is one of the most studied exportins involved in transporting critical cargoes, including tumor suppressor proteins like p27, p53, and RB1. Apart from the TSP cargo transport and its role in drug resistance, XPO1 inhibition results in retention of master transcription factors essential for cell differentiation, cell survival, and autophagy, rendering cells more susceptible to the effects of other antineoplastic agents, including targeted therapies. This review will dissect the role of XPO1 inhibition in hematological neoplasms, focusing on mechanistic insights gleaned mainly from work with SINE compounds. Future potential combinatorial strategies will be discussed.

## Introduction

Despite the progress made in identifying the most common molecular lesions in various hematological malignancies, the frequent lack of a key driver mutation, the complex interplay when multiple lesions are present, and molecular heterogeneity within coexisting sub-clones constitute a challenge for targeted therapeutics. For instance, despite drugs against actionable drivers in acute myeloid leukemia (AML) [1], the meaningful outcomes as seen with imatinib in chronic myeloid leukemia (CML) have not been replicated with regard to the long-term prognosis of new or refractory AML cases [2]. Additional targeted agents or other rationally applied drugs in combinatorial regimens constitute one of the most appealing approaches to overcoming treatment challenges. In this context, among various potential novel strategies, nuclear export inhibition is of particular interest. XPO1 inhibitors, with their inhibitory effects on various tumorigenic pathways, synergize with multiple antineoplastic agents used in hematological neoplasms and other cancers. Nuclear export as a therapy target has been a subject of basic and clinical research for a decade now. We explore its role as a backbone for combination therapeutic strategies. While preclinical studies in nuclear export inhibition have been successfully translated to bedside medicine in multiple myeloma (MM) and non-Hodgkin lymphoma (NHL), the same milestones have not been achieved in MDS and AML. Hence, we will focus more on the preclinical rationale for the combination strategy using XPO1 inhibition in MDS and AML specifically.

### Nucleocytoplasmic shuttling and the role of XPO1 in cancer

Nucleocytoplasmic shuttling is critical for the homeostasis of eukaryotic cells [3, 4] maintaining protein balance across the nucleus and cytoplasm essential for cell survival and death [4]. Neoplastic cells are heavily dependent on this process for their substantial metabolic demand. The ability to alter the nucleo-cytoplasmic traffic of essential cargo proteins creates opportunities to target various unique pathways implicated in carcinogenesis.

**Fig. 1:**
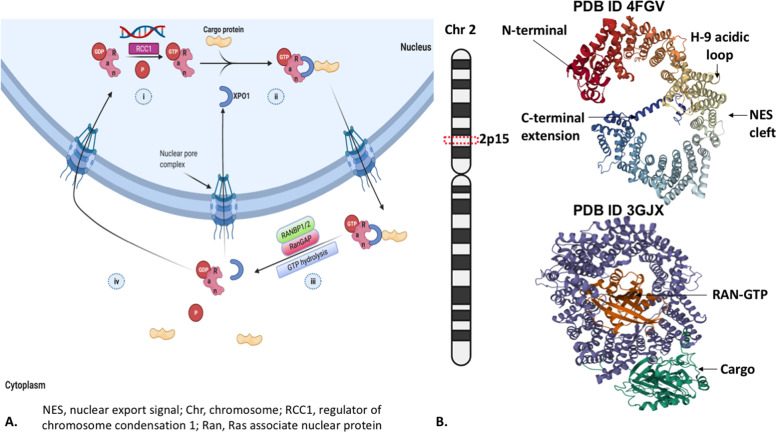
An illustrative picture of XPO1-dependant nuclear transport. **A** Nucleo-cytoplasmic shuttling process transports various cargo proteins critical for cellular functions through nuclear pore complexes (NPCs) that facilitate macromolecular exchange. (i), The chromatin-bound nucleotide exchange factor, the regulator of chromosome condensation 1 (RCC1) in the nucleus, aids the conversion of RanGDP to RanGTP. (ii), RanGTP binds with cargo protein-loaded XPO1, causing a conformational change to expose the binding site’s nuclear export signal (NES). The cargo protein’s leucine-rich NES domain interacts with the NES binding site of XPO1. The active complex containing RanGTP, XPO1, and the corresponding cargo protein is docked into the NPC and subsequently shuttled out of the nucleus. (iii), in the cytoplasm, the RanGTP-XPO1-cargo loaded complex is subjected to GTP hydrolysis with RanGAP (GTPase activating protein) along with other protein ligases, including RanBP1/2. First, it releases the RanGTP off the complex and, on hydrolysis, converts RanGTP to RanGDP, eventually maintaining a higher gradient of the latter in the cytoplasm. RanGTP less XPO1-cargo complex aids in releasing the cargo from XPO1. (iv), in the final step of the energy-dependent nucleocytoplasmic shuttling, XPO1 is relocated back to the nucleus. **B** Demonstrates XPO1 gene locus in chromosome 2, the crystal structure of free XPO1 protein, and the cargo loaded RAN-GTP state [obtained from PROTEIN DATA BANK: 10.2210/pdb4FGV/pdb and 10.2210/pdb3GJX/pdb].

Nuclear pore complexes are highly specialized structures embedded in the nuclear envelope and help transport various payloads across both directions [5]. Some are by simple diffusion, whereas others need an energy-dependent active transfer fueled by the RAN-GTPase system [4] (Fig. 1A). There are specialized protein receptors identified as importins and exportins maintaining the nucleo-cytoplasmic traffic. These receptors belong to the karyopherin family, which includes a broader subfamily of importin (IPO) α and IPO β. The exportin XPO1 (exportin 1)/CRM1(chromosome region maintenance 1), one of the most studied with respect to its function and implication in carcinogenesis, is classified under importin β superfamily of karyopherins [6, 7]. This shuttling process is navigated by recognizing specific amino acid sequences in the target proteins called basic residue-rich nuclear localization signal (NLS) and a leucine-rich nuclear export signal (NES) [8, 9]. Protein Data Bank provides crystal structures of NES-cargos bound XPO1 and ternary complexes of RanGTP/XPO1/cargos [10] (Fig. 1B).

Some of the cargoes exported through XPO1 are tumor suppressor proteins (TSPs) and cell cycle regulatory proteins, including p21, p27, p53, RB1, FOXO1, and Cyclin B1/D1. Although multiple studies have shown that upregulated expression of XPO1 is associated with poor prognosis in solid and liquid cancers [11–21], there is little data regarding the mechanism(s) leading to upregulated XPO1 expression. More functional information on XPO1 has been revealed by site-directed mutagenesis in the NES binding groove that can significantly reduce the XPO1 affinity to the cargos [22, 23]. In contrast, C-helix deletion in XPO1 increases its affinity to its cargos, restricting the cargo release rate [24, 25]. Further, the identification of recurrent missense mutations (*XPO1*^E571K^^,^^D724^^, and^ ^R749^) has exposed more mechanistic clues to the role of XPO1 in different cancers [26]. Particular hotspot mutations showed lineage specificity, especially in chronic lymphocytic leukemia (CLL), Hodgkin lymphoma, and primary mediastinal b-cell lymphoma. *XPO1*^E571K^ has been predominantly noted in NHL and Hodgkin lymphoma and less commonly in CLL. XPO1 mutations are postulated to alter the hydrophobic NES-binding groove, which might affect the open-closed equilibrium of the exporter, shape, and affinity of the binding groove to become preferential to certain export cargoes. The tumorigenic role of *XPO1*^E571K^ mutation was demonstrated in a Cre-inducible conditional knock-in mouse model where the mice developed a lethal b-cell malignancy similar to human CLL [27]. *XPO1*^E571^ has also been identified as a founder lesion in preneoplastic lymphocytes, where it can facilitate the acquisition of further genetic and epigenetic perturbations to transform to a malignant phenotype. Despite different explanations for possible functional impact for this mutation in various cancers, in vitro data on cell lines harboring the mutation did not show differential sensitivity to XPO1 inhibitors [28]. The prevalence of *XPO1*^E571K^ post-treatment in smaller studies has shown to be a negative prognosticator on survival and hence may serve as a biomarker for response but pending validation from larger studies [29].

More recently, it has become evident that XPO1 function is not just limited to the transport of TSP cargoes but also has a role in drug resistance, retaining master transcription factors essential for cell differentiation, cell survival, and autophagy. The mechanistic aspects of XPO1 inhibition using SINE (Selective Inhibitors of Nuclear Export) compounds can affect various processes that are associated with cancer cell proliferation, survival, adhesion, migration, or metastasis, and many of them are downstream of other known targets. The downstream action suggests that XPO1 inhibition could address the issue of pathway signaling redundancy and/or cross-talk contributing to drug resistance.

The introduction of SINE compounds and discernment of their critical role in nuclear transport has generated numerous studies focusing on the manipulation of carcinogenic pathways, including in AML. Unfortunately, these preclinical findings have not yet translated into success in early phase clinical trials in AML [30]. This is at least in part attributed to the expected off-target effects and resultant interruptions in therapy using first-generation SINE compounds rather than pharmacodynamic failure. Given the interest in combining SINEs with other therapies, the paradigm established in the treatment of multiple myeloma—namely using lower and more tolerable doses of SINEs in combination with other agents [31], may be an effective approach in other hematological neoplasms going forward.

### Evolution of XPO1 as a target

The concept of nuclear export inhibition dates back to the 1990s with the antitumor antibiotic leptomycin B (elastocin) [32, 33]. This compound irreversibly blocks Cys528 of XPO1 in the NES domain [32] (Fig. 1B). However, the phase 1 clinical trial with leptomycin B for refractory cancer patients was hampered by toxicities [34] attributed to the irreversible binding nature with the target. Development of other natural XPO1 inhibitors, including but not limited to leptomycin A, anguinomycins [35], and ratjadones A/B/C/D [36] produced similarly lackluster results.

Newer drug development strategies, namely Consensus-Induced Fit Docking (cIFD) methodology, established a novel approach to XPO1 inhibition with the development of the SINE class of XPO1 inhibitors [37]. These compounds, KPT-185, KPT-251, KPT-276, KPT-330 (selinexor), KPT-335 (verdinexor), KPT-8602 (eltanexor), and SL-801 (felezonexor), are reversible covalent small molecular inhibitors of XPO1 and hence expected to be less toxic than the previous generation compounds [38–44]. Because of the availability of modern improved XPO1 inhibitors, their logical applications in leukemias with specific mutations, myelodysplastic syndrome/acute myeloid leukemia (MDS/AML), non-Hodgkin’s lymphoma (NHL), and multiple myeloma (MM) may be more rational and involve combinations with old and new drugs.

### Role of XPO1 inhibition in driver mutations/pathways enriched in hematological neoplasms

**Fig. 2:**
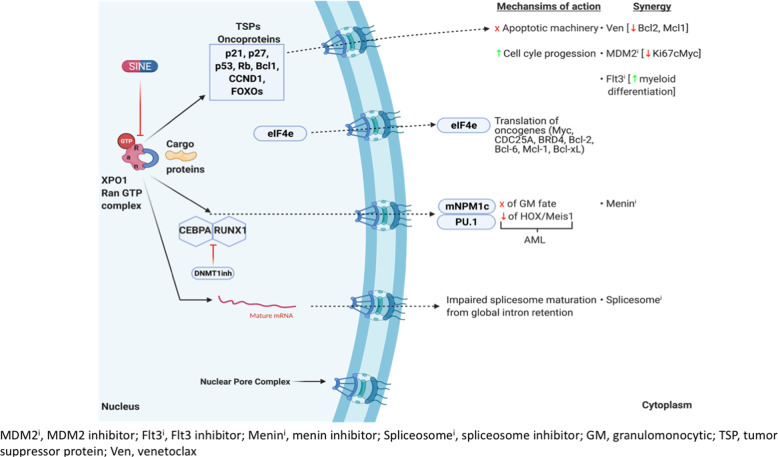
Mechanistic pathways interrupted by XPO1 inhibition and possible synergies. XPO1 transports several cellular protein cargoes and RNAs across the nuclear membrane into the cytoplasm. Important cargoes include tumor suppressor proteins like Rb1, p53, APC, and others to apoptosis. Potential synergy with anti-apoptotic inhibitors like Bcl2 (Ven, venetoclax) and MDM2 inhibitors is illustrated. Cell cycle growth regulators shuttled through XPO1 like p21, p27, and cyclin B1 maintain tumorigenesis. *FLT3*ITD, an oncogene in AML, can be inhibited by combining FLT3 and XPO1 inhibitor. NPM1 (Nucleophosmin) mutation translocate master transcription factor for monocytic differentiation PU.1 along with it to the cytoplasm when mutated. The NPM1c/PU.1 complex export dislocates it from CEBPA/RUNX1 transcription factor essential for granulomonocytic (GM) differentiation. XPO1 inhibition locks NPM1 within the nucleus enabling terminal monocytic differentiation. Upregulated Meis1/Hoxa9 in *NPM1* mutant AML is downregulated when NPM1 is retained within the nucleus and can synergize with menin inhibitors. CEBPA/RUNX1 interactome act as co-repressors on differentiation when unbound by NPM1/PU.1 complex and, when inhibited with DNMT1 inhibitors like decitabine, can aid GM terminal differentiation.

Inhibition of XPO1 with SINE compounds affects various known genetic drivers in hematological malignancies (Fig. 2). Some of these genetic drivers have currently approved targeted agents, while the rest are in the development pipeline.

#### TP53 and XPO1

TP53, a frequently mutated or deleted gene in hematological malignancies, is regulated by MDM2, an E3 Ubiquitin ligase [45]. XPO1 transports both p53 and MDM2, and previous preclinical studies have shown synergism in XPO1 and MDM2 inhibitors (selinexor and milademetan) in AML [15]. This effect was associated with upregulation of the TP53 pathway, inhibition of cMyc, and reduction of Ki-67 levels.

#### NPM1 and XPO1

*NPM1* (Nucleophosmin 1) is a frequently mutated gene in AML and confers an overall good prognosis [46, 47]. The insertion mutation in the highly conserved W288 or W290 residue of the C terminal end in *NPM1* causes a frameshift in the read that replaces an NLS (nuclear localization signal) with an NES. The net imbalance in the nuclear retaining signal shuttles out the mutant NPM1 (NPM1c) to the cytoplasm, which co-transports the master transcription factor PU.1 (SPI1) with it. The absence of PU.1 in the nucleus toggles the nuclear transcription collaborators *CEBPA* and *RUNX1* to act as co-repressors instead of activators of approximately 500 downstream genes essential for granulomonocytic differentiation (Fig. 2). XPO1 inhibition retains NPM1/PU.1 within the nucleus and thus activates monocytic fates [48]. In addition, NPM1c-mediated expression of the homeobox genes *HOX/Meis1* could explain the maintenance of the immature stem-like leukemic state in *NPM1* mutant AML. A phase 1 trial demonstrated safety with selinexor as monotherapy in the R/R (relapsed/ refractory) AML setting (NCT01607892) [49]. Only 1/5 of *NPM1* mutated patients in the cohort of 95 total AML patients in the study had a complete response (CR). A definite causal relationship between mutational status and response could not be ascertained. A phase II study [*N* = 42] of selinexor with 7 + 3 backbone for R/R AML patients found 3 out of 4 *NPM1* mutated patients in their trial with CR [50, 51]. XPO1 inhibition in *NPM1* mutated AML provides multiple targetable strategies either as monotherapy or in combination with, e.g., menin/KMT2A inhibitors [52], as it was shown to decrease expression of *HOX/Meis1* as well.

#### BCL2, MCL1, and XPO1

While BCL2 is often overexpressed in a range of hematological neoplasms, the anti-apoptotic dependence can be heterogeneous, especially in AML. Other anti-apoptotic proteins like MCL-1 and BCL-xL are often enriched in a mixed clone, and hence targeting BCL2 alone may be insufficient to eliminate the leukemic process. Because XPO1 regulates both BCL-2 and MCL-1 transport from the nucleus to the cytoplasm for translation [with a chaperon protein Leucine-rich PPR-motif-containing protein (LRPPRC)], SINE compounds/BCL2 inhibitor combination was shown to enhance cell death in in vitro experiments and patient-derived xenograft models in AML. SINE compounds perhaps prevent eIF4E (translation initiation factor) from augmenting BCL2 and MCL1 mRNA translation [53, 54]. The adjunct studies also support the ex vivo sensitivity of primary venetoclax refractory patient samples to the combination of SINE-Venetoclax. This combination was also tested to be synergistic in a different study with primary AML and DLBCL cells, and its action was mechanistically shown to be independent of P53 status [55]. It can potentially be a clinically significant finding, especially in 17p del CLL patients where anti-apoptotic factor MCL1 is degraded by other mechanisms.

#### FLT3 and XPO1

Generally, the *FLT3* gene mutation in AML is considered a poor prognosticator though the allelic frequency could partly impact this negative correlation’s strength [56, 57]. Dual targeting of XPO1 and *FLT3* increased pro-apoptotic signal by retaining TSPs in the nucleus [58]. The combination was synergistic in vitro and in in vivo human xenograft *FLT3* mutated mouse models. Myeloid differentiation of the *FLT-ITD* clone was enhanced by co-targeting XPO1 and *FLT3* instead of targeting either alone. An early-phase study with R/R *FLT3* mutated AML patients [58] [50% exposed to prior FLT3 inhibitors] showed a sustained CRi/CRp (complete remission with incomplete hematological recovery/complete remission with incomplete platelet recovery) in 29% (4/14), and two additional patients (14%) showed more than 50% blast reduction. Moreover, the responding patients were also MRD (measurable residual disease) negative for *FLT3-ITD* by RT-qPCR.

#### SF3B1 and XPO1

A recent post-hoc analysis from a phase 2 study in MDS [59] and oligoblastic AML (20–30% blasts) patients refractory to hypomethylating agents (HMAs) demonstrated patients with the canonical splicing factor mutation in *SF3B1* responding significantly better to selinexor. While *SF3B1* is mostly a good prognosticator in MDS, in this study, the *SF3B1* patients had high-risk disease by IPSS-R. Inhibition of XPO1-mediated RNA transfer required for spliceosome machinery’s maturation may produce synthetic lethality in *SF3B1* mutant disease, and this selective sensitivity of *SF3B1* to XPO1 inhibition merits further research.

#### Epigenetic perturbations and XPO1 inhibition

Hypomethylating agents affecting epigenetic perturbations in myeloid neoplasms have been combined with other novel agents. Recent preclinical studies, both in vitro and in vivo, showed decitabine priming to enhance the antileukemic effects of selinexor [60]. The synergism was more evident at lower doses of selinexor. It hence could avoid the potential untoward toxicities of higher doses of selinexor used in other studies as a single agent.

### Overcoming drug resistance with XPO1 inhibition

#### Imatinib/dasatinib (Fig. 3)

Protein mislocalization is one of the critical factors recognized in oncological drug resistance. Despite successes in treating CML with tyrosine kinase inhibitors (TKI), some patients still develop TKI resistance. Studies using TKI with leptomycin B (XPO1 inhibitor) demonstrated BCR-ABL trapping within the nucleus, causing irreversible and complete destruction of the BCR-ABL clone [61]. Ex vivo studies using human CML samples also corroborated those findings [62]. Targeting nuclear export can eradicate CML clones in scenarios, especially where there are TKI resistant mutations. The implications are broader and perhaps be applicable in all ph+ leukemias and CML blast crisis.

**Fig. 3:**
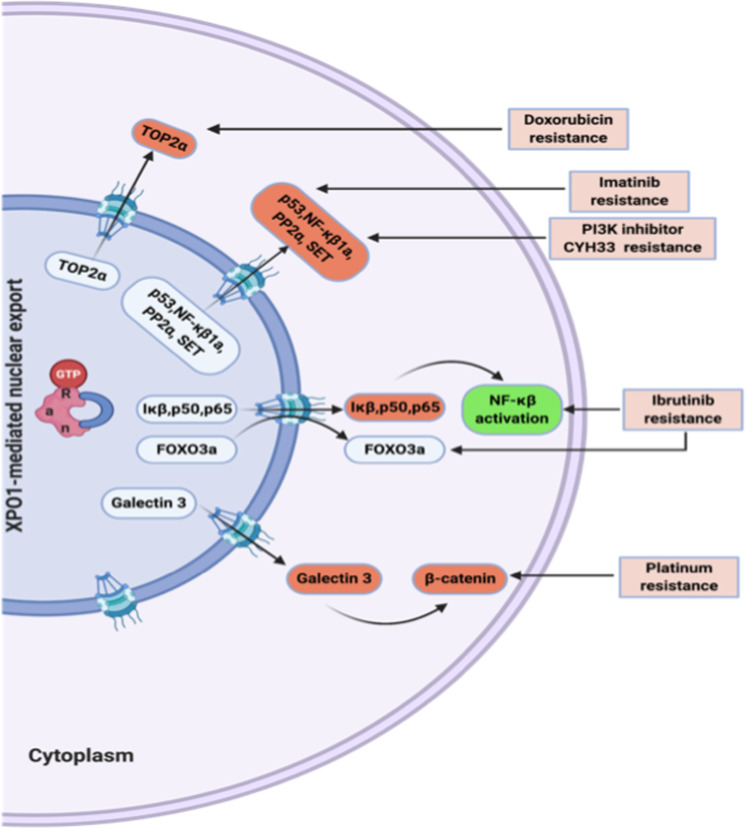
Mechanisms of XPO1 mediated emergent drug resistance pathways. Increased XPO1 expression mediates nucleocytoplasmic displacement and inactivation of tumor suppressor proteins, leading to tumorigenesis as well as emergent drug resistance. Cytoplasmic dislocation of P53 is implicated in imatinib’s acquired drug resistance and PI3K inhibitor CYH33. Mislocalized topoisomerase 2α (TOP2A) to the cytoplasm is linked to doxorubicin resistance. Ibrutinib resistance is associated with XPO1 mediated nuclear export of inhibitors of NF- κB (IκB), P50, and P65, leading to activation of NF-κB signaling pathway. The nuclear export of FOXO3A is illustrated in acquired ibrutinib resistance, which XPO1 inhibitors can overcome. Platinum resistance is linked to β-catenin, which is regulated by the XPO1 mediated cytoplasmic displacement of Galectin 3.

#### Bortezomib/carfilzomib

Acquired resistance to proteasome inhibitors (PI) is common in MM. A proteomics-based approach [63] revealed and validated one hundred and twelve regulatory proteins differentially expressed with bortezomib resistance in MM, and many of them interacted with XPO1 [63] (CSE1, DYNLL1, HSPA14, NUP88, NUP50, RAD21, RCC2, RANBP2, SMC1A, and TPR). XPO1 knockdown in myeloma cell lines reestablished sensitivity to proteasome inhibitor. Thus, XPO1 inhibition could help overcome drug resistance in MM. Preclinical studies tested PI-resistant myeloma cell lines and patient-derived xenograft models treated with selinexor/bortezomib or carfilzomib [64] resensitized resistant cells by diminishing NFκB transcriptional activity.

#### Anthracyclines and cytarabine

AML can develop resistance to Topo II inhibitors (anthracyclines and etoposide) when Topo IIα is mislocalized to the cytoplasm. This displacement is XPO1 mediated, and SINE compounds have been shown to retain Topo IIα back in nucleus reestablishing sensitivity to the Topo II inhibitors [65]. In a head and neck cancer model, XPO1 inhibition was shown to prevent cytoplasmic mislocalization of the transcriptional repressor E2F7 from its nuclear transcriptional activator E2F1 counterpart (for drug-resistant genes) and thus overcome anthracycline drug resistance [66].

#### Ibrutinib

Selinexor has single-agent activity in CLL cells and, when combined with ibrutinib, cause synergistic cytotoxicity in primary CLL cells [67]. Selinexor is also effective in vitro in CLL cells harboring a resistant BTK C481S mutation and in vivo in the ibrutinib-refractory mice model. Mechanistically, ibrutinib resistant cells decrease FOXO3a levels in the nucleus, and selinexor could overcome ibrutinib resistance by retaining FOXO3a within the nucleus [68]. It is noted that failure to inhibit NFκB transcriptional signature is associated with ibrutinib resistance in MCL cell lines [69]. Selinexor retains IκB, P65, and P50 within the nucleus and in the bound state; P65 and P50 are inactive in DNA binding. Hence, targeting downstream of BCR signaling on NFκB with selinexor could negate the acquired upstream resistance mutations.

### Clinical studies in MDS/AML with SINE compounds—an overview

Table 1 provides a list of MDS and/or AML trials involving XPO1 inhibitor therapy. Below, we provide additional details regarding some of these studies.

**Table 1:** Clinical studies in acute myeloid leukemia and myelodysplastic syndromes.

Study and patient characteristics	Intervention	Selinexor dosing (PO)	Outcomes	Grade 3–4 TEAE
NCT01607892 Phase 1 95 pts. ≥65 years with R/R AML [49]	3 + 3 study design Selinexor	Dosing ranges studied: 16.8 to 75 mg/m^2^	CR/CRi 7/81 (9%), MLFS 1/81(1%), PR 3(4%) and ORR 14%. RP2D 60-mg flat dose (∼35 mg/m^2^)	Nausea 5% (5/95), vomiting 5%, fatigue 13%, thrombocytopenia 20%
NCT02228525 Phase 2 single arm 25 pts. ≥18 years with high-risk MDS or oligoblastic AML refractory to HMAs [59]	Selinexor	Dosing amended after first 3 patients: flat dose 60 mg twice weekly for 2 weeks with 1 week off	ORR 26% (6/23) with 6 in marrow CR. SD in 12 (52%)	Hyponatremia 20% (5/25), neutropenia 20% and thrombocytopenia 33%
NCT02835222 Phase 2 Randomized 28 pts., ≥60 years N/D de novo AML [100]	3:1 7 + 3+Selinexor or 7 + 3f/b high dose Ara-C ± Selinexor. Selinexor maintenance in intervention arm	Sixty milligram on days 1, 3, 8, 10, 15 and 17 during I/C and on days 1 and 8 every 21 days during maintenance	27/28 pts. evaluable. Std arm—CR/CRi 43% (3/7). Selinexor arm—CR/ CRi 86% (18/21). 7/21 post selinexor transplanted	33% (7/21) in the selinexor arm had prolonged thrombocytopenia (>4 weeks). 60-day mortality similar in both arms (10%)
NCT02093403 Phase 1 20 pts. with ND AML, 5 pts. ≥60 years with R/R AML [70]	Selinexor and decitabine	Four dose ranges between 23–55 mg/m^2^	ORR 40% (10/25). 5 CR and 3 Cri. 60 mg flat dose twice-weekly for 2 weeks after HMA better tolerated	Anemia 23 (92%) and thrombocytopenia 25 (100%). Hyponatremia in 17 (68%)
NCT02403310 Phase 1 21 pts. ≥60 years with ND poor-risk AML [74]	Selinexor + daunorubicin and cytarabine (7 + 3)	Two doses: 60 mg and 80 mg given twice weekly for 3 weeks	8 CR and 2 CRi and ORR of 53% (10/19). Median OS 10.3 mo. with median f/u of 28.9mo	Febrile neutropenia (67%), diarrhea (29%), hyponatremia (29%), and sepsis (14%)
NCT02249091 Phase 2 42 pts. with R/R AML, 5 pts. ≥60 years with R/R AML [50]	Selinexor + idarubicin and cytarabine (7 + 3)	Two cohorts: 40 mg/m^2^ twice weekly for 4 weeks and 60 mg flat dose twice weekly 3 out 4 weeks	20 CR/CRi and ORR 47·6%. 35·7% (15/42) were transplanted	Febrile neutropenia 67% (28/42), thrombocytopenia 62% (26/42), anemia 57% (24/42), diarrhea 50% (21/42)
NCT02485535 Phase 1 12 pts. as maintenance after allogeneic SCT for MDS and AML [76]	Selinexor	Between 60 and 100 days after allo-SCT dosed as 60 mg or 80 mg weekly for 4 weeks: up to 12 cycles or if no relapse	4/12 pts. completed 12 cycles. Median PFS and OS 775 days and 872 days resp. 3/4 pts. didn’t relapse	Any grade AE includes nausea (91.7%), anorexia (66.7%), fatigue (66.7%), vomiting (25%), and diarrhea (18%)
NCT02649790 Phase 1/2 20 pts. ≥60 years of age with high-risk MDS [96]	Eltanexor	Dose levels: 10 and 20 mg daily for 5 consecutive days/week in a 28-day cycle	Marrow CR 29% (4/14), 43% SD (6/14) and 29% PD (4/14). DCR 71%	Any grade AE nausea (53%), low appetite (33%), diarrhea (27%), thrombocytopenia, anemia, neutropenia, vomiting, and dysgeusia (20% each)
NCT02530476 Phase 1b 14 patients, FLT3-ITD and -D835 MT R/R AML [prior FLT3i allowed] [95]	Selinexor + Sorafenib	Dose escalation phase: 40, 60, and 80 mg twice weekly [28-day cycle]	CRp/Cri 29% (4/14). Blast reduction >50% in 2/14 (14%). 6/11 (55%) prior FLT3i exposed pts. responded	Bleeding (36%), febrile neutropenia 28%, and pneumonia (21%)

*AML* acute myeloid leukemia, *CR* complete response, *CRi* complete response with incomplete hematological recovery, *CRp* complete response with incomplete platelet recovery, *DOR* duration of response, *DCR* disease control rate, *f/b* followed by, *HMA* hypomethylating agent, *MDS* myelodysplastic syndrome *MLFS* median leukemia free survival, *MTD* maximum tolerated dose, *N/D* newly diagnosed, *OS* overall survival, *ORR* overall response rate; *PR* partial response, *PD* progressive disease, *PFS* progression free survival, *RP2D* recommended phase II dose; *R/R* relapse/refractory, *SD* stable disease, *SCT* stem cell transplant, *Std* standard, *TEAE* treatment emergent adverse events.

Selinexor monotherapy is safe and, in some cases, efficacious in patients with advanced hematological malignancies based on a phase 1 study [49]. After a robust anti-leukemic activity in AML preclinical models, 95 patients with R/R AML not candidates for chemotherapy were enrolled in this trial [NCT01607892]. In a heavily pretreated population (>3 lines of prior therapy), 7 out of 81 evaluable patients (14%) responded [five complete responses (CR) and two complete responses with incomplete hematological recovery (CRi)]. In responding patients, the median progression-free survival (PFS) and overall survival (OS) were 5.1 and 9.7 months, respectively. Stable disease (SD, ≤50% increase in BM blasts from baseline) was noted in 65% of patients. While no cytogenetic or molecular features correlated with response, patients with low blast count or hypoproliferative AML responded better. A separate single-arm phase II study examining selinexor as therapy for patients with HMA refractory myelodysplastic syndrome, or oligoblastic acute myeloid leukemia [59] showed selinexor to have promising activity. 6 of 23 evaluable patients (26%) attained a marrow CR, and 12 patients (52%) had SD. Interestingly, the post-hoc analysis revealed patients with disease harboring *SF3B1* mutations had a higher likelihood of responding [59].

After preclinical work showed selinexor had robust activity in AML post decitabine priming [60], a phase I dose-escalation study assessing selinexor in combination with HMAs in older (age ≥ 60) relapsed/refractory (R/R) and newly diagnosed (N/D) AML patients [*N* = 25] [70] was done. More than half were heavily pretreated (≥3 lines of prior therapy). The ORR was 40% (10/25 patients). Five of the responders achieved a CR, and another three had attained a CRi. Selinexor was better tolerated at 60 mg (flat dose) given twice a week for 2 weeks after decitabine treatment. Selinexor was also combined with other DNA-damaging conventional chemotherapies in several small early phase studies that included N/D poor-risk AML or R/R disease [50, 71–74]. A single-arm phase 1 study of selinexor with the traditional 7 + 3 regimen [cytarabine and daunorubicin] for N/D poor-risk AML patients [74] showed a CR/CRi of 53% (10/19: 8CR, 2CRi). More than a third of patients were alive at the median follow-up of 28.9 months. The study established the safety of adding selinexor to the conventional 7 + 3 backbone, with a response rate at the upper end of the historically reported range for poor-risk N/D AML (20–50%) [75]. Although some patients with AML and MDS are cured with allogeneic stem cell transplant, the majority of these high-risk patients ultimately relapse. Maintenance selinexor post hematopoietic stem cell transplant for MDS and AML patients delayed relapse in a small study [76]. Three out of the four patients who completed all the 12 cycles had not relapsed at the time of the published report.

In conclusion, selinexor has demonstrated potential as monotherapy and in combination with other novel agents and chemotherapy in MDS/AML. Improved response rates amongst patients with low blast count disease argue its applicability in high-risk MDS patients rather than in high blast count AML, where the biology may differ. Dose and/or schedule modification to improve overall tolerance of selinexor-based therapy is perhaps a clinically meaningful strategy for older and less fit AML/MDS patients with limited treatment options. Also, further clinical work assessing the activity of XPO1 inhibitors in patients with specific molecular features (e.g., *SF3B1*) and/or clinical parameters is needed to determine whether any particular subgroup of patients stands to benefit more from these drugs. Selinexor as a maintenance strategy in the post-stem cell transplant or other therapies seems reasonable and needs further exploration.

### Clinical studies in non-Hodgkin’s lymphoma (Table 2)

Despite the availability of cellular therapies for patients with R/R NHL, overall such patients have a poor prognosis [77, 78]. Moreover, only a fraction of patients qualify for such intensive treatment. Hence, there is an unmet need in developing novel therapeutics for this patient population.

**Table 2:** Clinical studies in non-Hodgkin’s lymphoma.

Study and patient characteristics	Intervention	Selinexor dosing (PO)	Outcomes	Grade 3–4 TEAE
NCT01607892 Phase 1 79 pts. with R/R NHL including DLBCL, RT, MCL, FL, MZL, and CLL [79]	Selinexor	Dose-escalation phase: 3–80 mg/m^2^ in 3 or 4 week cycles. Dose-expansion phase: 35 or 60 mg/m^2^	ORR 31% (22/70) with 18 PR and 4 CR; DCR for DLBCL 51% (21/41) including the 4 CR	Thrombocytopenia (47%), neutropenia (32%), anemia (27%), leukopenia (16%)], fatigue (11%) and hyponatremia (10%)
NCT02227251 Phase 2b (SADAL) 267 pts. with R/R DLBCL (48 excluded for enrollment before ver. 6.0 of protocol) [80]	Selinexor	Hundred milligram twice weekly dosing cohort discontinued (*N* = 92). Sixty milligram twice daily until PD, death, or intolerance to the drug	ORR 28% (36/127) with 15 (12%) CR and 21 (17%) PR; Median DOR for responders 23.0 mo. (95% CI 10.4–23.0)	Thrombocytopenia (46%), anemia (22%), neutropenia (25%), fatigue (11%), and hyponatremia (8%)
NCT02303392 Phase 1 33 pts. with CLL, RT, DLBCL, and MCL [82]	Selinexor and ibrutinib	Selinexor 1–2 times weekly (3 weeks of a 4-week cycle) and ibrutinib 420 mg daily starting Cycle 1 Day 8. Treatment repeated until PD, intolerance, or death	MTD was 40 mg weekly of selinexor along with ibrutinib 420 mg; ORR 33% (11/33) and DCR 81% (27/33)	Neutropenia 9%, anemia 3%, and weight loss 5%. Five treatment unrelated deaths
NCT03147885 Phase 1 12 pts. with ND NHL [83]	Selinexor with R-CHOP	R-CHOP given with weekly Selinexor for six cycles f/b maintenance Selinexor for 12 months	MTD NR. RP2D 60 mg weekly. ORR 100% and CRR 90% (median f/u 476 days)	Infrequent nausea (2/6 in 80 mg cohort) and fatigue (1/6 in 60 mg cohort)

*CLL* chronic lymphocytic leukemia, *CR* complete response, *DOR* duration of response, *DCR* disease control rate, *DLBCL* diffuse large b-cell lymphoma, *FL* follicular lymphoma, *MCL* mantle cell lymphoma, *MZL* marginal zone lymphoma, *MTD* maximum tolerated dose, *ND* newly diagnosed, *NHL* non-Hodgkin lymphoma, *NR* not reached, *OS* overall survival, *ORR* overall response rate, *PR* partial response, *PD* progressive disease, *PFS* progression free survival, *RT* Richter’s transformation, *RP2D* recommended phase II dose, *R/R* relapse/refractory, *TEAE* treatment emergent adverse events.

Selinexor as monotherapy was tested in a phase 1 trial [79] with seventy-nine non-Hodgkin’s lymphoma (NHL) patients [chronic lymphocytic leukemia (CLL), diffuse large B-cell lymphoma (DLBCL), follicular lymphoma (FL), mantle cell lymphoma (MCL), and Richter’s transformation (RT)]. Among the 70 evaluable patients, 22 (31%) had an objective response, including 18 partial responses (PR) and 4 CR. All four CRs were seen in the DLBCL patients [10% (4/41)]. Including SD, the DLBCL cohort had a disease control rate (DCR) of 51% [21/41]. Six patients who showed some response were either double or triple hit lymphomas. There was 1 CR and 2 PR in this subgroup of aggressive histology. The FDA recently approved selinexor for R/R DLBCL patients [≥2 lines of prior treatment] after a subsequent multinational open-label phase 2b study, SADAL [80, 81], showed an ORR of 28% (36/127 evaluable patients), including 15 CR (12%), 21 PR (17%), and 11 SD (9%). More patients in the germinal center B-cell (GCB) cohort responded [ORR 34% (20/59)] than in the non-GCB cohort [21% (13/63)]. In a phase 1 trial of selinexor plus ibrutinib [82] involving 33 patients with R/R CLL/NHL [16 CLL, 8 Richter’s transformation, 6 DLBCL, and 3 MCL], disease control was achieved in 81%. Among them, 33% had CR or PR, and the remaining maintained SD. Prior ibrutinib exposure was best associated with SD, whereas ibrutinib not-exposed patients showed CR or PR. Of note, the two CLL pts with known BTK mutation responded to the combination treatment. Also, one of the six RT patients who responded had a CR. After a median follow up of 5.3 months (1.2–22.5 months), median PFS/OS for CLL and NHL patients were 8.9 (95% CI: 4.6–NR)/NR (95% CI: 15.4–NR) and 2.7 (95% CI: 0.7–NR)/5.4 (95% CI: 2.6–NR) mo., respectively.

The clinical feasibility of combining R-CHOP with selinexor in the frontline management of newly diagnosed NHL was shown in a phase 1b study with durable efficacy and a tolerable safety profile [83]. In that study (*n* = 12) with ten evaluable patients for a response, ORR and CRR were reported as 100% and 90%, respectively, after a median follow-up of 476 days. The RP2D of selinexor was 60 mg weekly. A separate phase Ib trial studying the combination of venetoclax and selinexor [NCT03955783] is currently recruiting for a total enrollment of 78 patients with high-risk R/R hematologic malignancies [AML and DLBCL] [Supplemental Table 1] is expected to report results by 2023.

In summary, selinexor is promising both as monotherapy and in combination therapy effective in different b-cell lymphomas. The robust RR in newly diagnosed NHL when selinexor is given in combination with upfront standard of care treatment supports the rationale for further testing, which is ongoing. The preferential response seen in aggressive R/R NHL makes it more attractive to add selinexor in different combination regimens.

### Clinical studies in multiple myeloma (Table 3)

Novel agents used in treating MM have changed the dismal outlook of an aggressive disease to an illness that can be managed effectively. Despite these advances, the disease is still not curable. Hence, there is a salient need to probe for targeting unique pathways with novel agents.

**Table 3:** Clinical studies in multiple myeloma.

Study and patient characteristics	Intervention	Selinexor dosing (PO)	Outcomes	Grade 3–4 TEAE
NCT02186834 Phase 1 11 pts. with R/R MM (≥2 prior treatments including lenalidomide and a proteasome inhibitor) [101]	Selinexor and dexamethasone with doxorubicin	Loading phase dosing for selinexor: 40 mg/m^2^, 60 and 80 mg	20% VGPR (2/10), 20% PR (2/10), 20% MR (2/10), 30% SD (3/10) and 1 PD	Hyponatremia 54%, thrombocytopenia 54%, neutropenia 54% and anemia 45%
NCT01607892 Phase 1 25 pts. in dose escalation and 59 pts. in dose expansion phase with R/R MM (six median prior therapies) [84]	Selinexor and dexamethasone	Dose escalation phase: 8 or 10 times (3–60 mg/m^2^) in every 28-day cycle; Dose expansion phase: 45 or 60 mg/m^2^ with d, twice weekly in 28-day cycles, or 40 or 60 mg flat dose without d in 21-day cycles	Single-agent ORR: 4% (2/57) and CBR 21% (12/57); 45 mg/m^2^ selinexor + dexamethasone: ORR 50% (6/12) and CBR 58% (7/12). RP2D 45 mg/m^2^	Thrombocytopenia (45%), anemia (23%), neutropenia (23%), hyponatremia (26%), and fatigue (13%)
NCT02336815, part I STORM Phase 2b 79 pts. with R/R MM (7 median prior therapies) [88]	Selinexor and dexamethasone	Selinexor 80 mg twice weekly on a 28-day cycle	ORR 21% and CBR 33%; high-risk cytogenetics: ORR 35% and CBR 53%; median PFS and OS: 2 and 9.3 months respectively	Thrombocytopenia 59%, anemia 28%, neutropenia 23%, hyponatremia 22% and fatigue 12%
STORM Part 2 Phase 2b confirmatory 122 pts. with R/R MM (triple class refractory) [89]	Selinexor and dexamethasone	Selinexor 80 mg with dexamethasone (20 mg) twice weekly for 4 weeks	ORR 26% (32/122; 2 stringent CR, 6 VGPR, and 24 PR) and CBR 39% (48/122). Median DOR, PFS, and OS: 4.4, 3.7, and 8.6 months, respectively	Thrombocytopenia (59%), anemia (44%), neutropenia (21%) and (22%) hyponatremia
NCT03110562^a^ Phase III BOSTON 402 pts. R/R MM (1–3 lines of prior therapy) [31]	Selinexor ± bortezomib and dexamethasone	Selinexor 100 mg once weekly for 5 weeks along with weekly 1.3 mg/m^2^ bortezomib and dexamethasone (20 mg) twice weekly	SVd vs. Vd: median PFS 13.93 months vs. 9.46 months [HR 0.70] after a median follow-up of 13.2 months and ORR 76.4% vs. 62.3% [OR 1.96]	SVd vs. Vd: Thrombocytopenia (39% vs. 17%), fatigue (13% vs. 1%) and anemia (16% vs. 10%)
NCT02199665 Phase 1 21 pts. with R/R MM (four lines of prior therapy) [93]	Selinexor + carfilzomib and dexamethasone	Dose level 1: 30 mg/m^2^; dose level 2–5: 40 and 60 mg twice-weekly along with carfilzomib 20/27 mg/m^2^, and dexamethasone 20 mg	ORR 38%, CBR 67%; median PFS and OS 3.7 and 22.4 months, resp.	Thrombocytopenia (71%), anemia, lymphopenia, and neutropenia (33% each resp.).
NCT02343042 STOMP Phase 1/2b 34 pts. with R/R MM (3 median prior therapies) [94]	Selinexor + daratumumab and dexamethasone	Selinexor 100 mg weekly or 60 mg bi-weekly with dexamethasone 40 mg (single or divided doses) in 28‐day cycles	ORR 69% (22/32), CBR 81% (26/32). DOR in responders 11.4 months	Thrombocytopenia (57%), anemia (32%), neutropenia (26%), fatigue (18%) and hyponatremia (12%)
NCT02649790 Phase 1/2 39 pts. with R/R MM (7 median prior therapies) [102]	Eltanexor + dexamethasone	Dose escalation phase 5 to 60 mg daily for 5 days along with dexamethasone	ORR 21% (7/34) and CBR 47% (16/34); RP2D was 20 mg daily × 5 days/28-day cycle	Thrombocytopenia (56%), neutropenia (26%), anemia (13%), and hyponatremia (8%)

*CBR* clinical benefit rate, *DOR* duration of response, *HR* hazard ration, *MM* multiple myeloma, *N/D* newly diagnosed, *OS* overall survival, *ORR* overall response rate, *PFS* progression free survival, *RP2D* recommended phase II dose, *R/R* relapse/refractory, *TEAE* treatment emergent adverse events.

^a^Recruiting

The single-agent activity of selinexor in heavily pretreated MM patients [84] was suboptimal, with 21% of patients achieving a minor hematologic response or better but an objective response rate (ORR, defined as a partial response or better) of only 4%. However, the addition of corticosteroids substantially increased the ORR to 50%. The ORR for single-agent glucocorticoids in a heavily pretreated population is historically in the range of 6–10%, strongly suggesting the combination of selinexor and dexamethasone synergize [85]. Preclinical evidence supported selinexor in combination with steroids to potentiate apoptosis in myeloma cells, possibly from repressed mTORC1 signaling [86, 87].

The FDA approved selinexor for R/R MM after the subsequent phase 2b STORM trial (NCT02336815, part I, Selinexor Treatment of Refractory Myeloma) [88] demonstrated an ORR of 21% and a clinical benefit rate (CBR = VGPR, very good partial response + PR + MR, minimal response) of 33%. Of note, 40% (31/79) of the study population were penta-refractory, including resistance to an anti-CD38 monoclonal antibody. High risk cytogenetic group [t(4;14), t(14;16) and del 17p] had an ORR of 35% and a CBR of 53%. The median PFS and OS were 2.3 and 9.3 months, respectively. The second part of the STORM trial [89] was a confirmatory study that enrolled a more homogenous cohort of R/R MM patients (median number of prior treatments = 7). More importantly, these patients were penta-exposed but triple class refractory (at least one immunomodulatory agent, one proteasome agent, and anti-CD 38 antibodies). The ORR was 26% (32/122; 2 stringent complete responses, 6 VGPR, and 24 PR), and the CBR was 39% (48/122). The two patients with a history of CAR-T cell therapy also had a PR. The median duration of response, PFS, and OS were 4.4 (95% CI 3.7–10.8), 3.7 (95% CI 3.0–5.3), and 8.6 (95% CI 6.2–11.3) months, respectively. A post hoc analysis [90] showed the combination to be effective for plasmacytomas. Approximately less than half of the plasmacytoma patients (44%, 7/16) in the study with follow-up assessments showed complete resolution or decreased extramedullary disease size or metabolic activity. Based on this data, the FDA approval specifies that MM patients treated with selinexor must be refractory to at least two PIs, two immunomodulatory agents, and an anti-CD 38 monoclonal antibody are eligible to receive selinexor plus dexamethasone.

A phase 1b/II trial [STOMP] established the safety of using the triplet combo (bortezomib, dexamethasone, and selinexor) to treat R/R MM [*N* = 42] [91]. In this trial, the ORR was 63% and approximately half of the patients previously documented to be refractory to proteasome inhibitor-based therapy responded. The median PFS was 9.0 months. Of note, there was a lower incidence of neuropathy, an important dose-limiting toxicity of bortezomib. In the phase 3 (BOSTON) [31] open-label study, 402 MM patients who had received 1–3 lines of prior therapy were randomized to bortezomib and dexamethasone with (*N* = 207) or without selinexor (*N* = 195). The triplet arm with selinexor had a superior median PFS of 13.93 months (95% CI 11.73–not evaluable) vs. the doublet with 9.46 months [(8.11–10.78); HR 0.70] after a median follow-up of 13.2 months (IQR 6.2–19.8). The triplet arm had a significantly superior ORR as well compared to the doublet arm [76.4% (95% CI 69.8–82.2) vs. 62.3% (55.3–68.9); odds ratio (OR) 1.96] and reported less frequent ≥ Grade 2 peripheral neuropathy [41 (21%) vs. 70 (34%); odds ratio 0.50 (95% CI 0.32–0.79), *p* = 0.0013]. Though nuclear export inhibitors may reverse inflammatory demyelination [92], in this case, the less frequent bortezomib dosing (once weekly) in the triplet arm seemed a likely reason for the lower incidence of neuropathy. The favorable efficacy, even in bortezomib refractory patients, led the FDA to approve the combination of selinexor, bortezomib, and dexamethasone to treat adult MM patients in the first relapse. The ORR (38%)and CBR (67%) were comparable when selinexor was combined with a different proteasome inhibitor carfilzomib in a phase 1 study [93]. When given in combination with daratumumab and dexamethasone in a phase 1/2b study [94] with heavily treated R/R MM patients [*N* = 34 with more than 2/3rds of the patients (71%) having had autologous stem cell transplant], the ORR and CBR were 69% and 81%, respectively (compared to 73 and 87% in the daratumumab naïve arm).

Overall, although selinexor has minimal single-agent activity in myeloma but regimens using the drug in combination with steroids and other novel agents result in far more efficacious clinical utility both in R/R and newly diagnosed MM. It also enables dose reductions of other agents used in the treatment of MM, ultimately improving the tolerability of the backbone regimen. Hence, future work will focus on using selinexor as a synergistic partner drug to other established and experimental anti-myeloma agents.

### Treatment-emergent adverse events and newer generation SINE compounds (Tables 1–3)

Hematological toxicities are common with selinexor and often require closer monitoring, especially in heavily pretreated patients with baseline cytopenias [31, 49, 51, 59, 76, 77, 80, 95]. This is likely from the poor bone marrow reserve. The most common non-hematological toxicities include fatigue, hyponatremia, nausea, vomiting, and diarrhea which are a class effect of the medication. Importantly, eltanexor (KPT 8602), a second-generation SINE compound with a similar pharmacokinetic profile as selinexor, has an improved side effect profile compared to selinexor, likely from far lower blood-brain-barrier penetration. Further, preclinically, eltanexor is efficacious against leukemia-initiating cells and AML blasts in vivo [39]. A phase 1/2 study [96] found single-agent eltanexor to be effective in elderly HMA-refractory HR-MDS (14 evaluable patients), where ten had meaningful responses with a disease control rate of 71% [four marrow (m) CR (29%) and six SD (43%)], and the remaining progressed (29%). Eltanexor was evaluated for safety and tolerability in a separate phase 1 study (*N* = 39) involving R/R MM patients (median prior therapies = 7). The ORR was modest [21% (7/34)] among the 34 evaluable heavily pretreated population, and the CBR was 47% (16/34). The most frequent grade 3–4 TEAEs were hematological; thrombocytopenia 56% (22/39), neutropenia 26% (10/39), anemia 13% (5/39), and hyponatremia 8% (3/39). The GI and other constitutional side effects were minimal and felt to be less problematic than that seen in other trials in which selinexor was the SINE compound used.

More recent studies in various blood cancers have demonstrated a strategy to mitigate these adverse effects by using selinexor in combination with salicylates [97]. The combination was shown to be not toxic for normal cells. Much of the class toxicity is dose-dependent. Therefore, if novel combinations allow using lower doses of selinexor as in some MDS and lymphoma trials, it could help extract the maximum target efficiency of the drug class, alleviating the emergent side effects. Perhaps, one could also hypothesize that short interrupted use of SINE compounds to synergize with other novel agents to mitigate the class’s treatment-emergent side effects could be an approach to test.

### Novel synergies in the horizon and conclusion (Fig. 2)

XPO1 inhibition has evolved as a novel target in cancer therapeutics with potential utility across different cancer types. The ability to cross-talk with different interactomes makes it an ideal synergy partner with other targeted therapeutics. Many of these mechanisms of actions of SINE compounds are downstream of activated pathways in cancer and hence can theoretically work in additional upstream acquired target drug resistance. Several preclinical studies have demonstrated this role in a wide variety of solid and hematological neoplasms, including AML/MDS. Combining MDM2 inhibitors and selinexor has been tested and possibly a strategy that can effectively mitigate *TP53* mutations in different malignancies. SINE compounds are active in *NPM1* mutated AML, ultimately altering the downstream HOX/MEIS program that maintains the leukemogenesis and could be potentially combined with other targeted agents like menin inhibitors. SINE compounds retain pro-apoptotic factors in the nucleus, making it ideal to be combined with Bcl2 inhibitors, which may also prevent resistance. Spliceosome inhibitors, though, looked promising in preclinical studies [98] showed an only mediocre response in the clinical trial [99]. Thus, the SINE compound could be combined with spliceosome inhibitors for its role in global intron retention, an action common to both classes of drugs.

However, striking a balance between maximizing the anti-cancer activity of SINE compounds while minimizing toxicity remains a challenge. Second-generation SINE compounds appear to have an improved therapeutic index compared to earlier agents. Still, even with first-generation compounds, there have been some successes in the clinic already, with the FDA recently approving selinexor-based therapy in the second-line setting for relapsed MM patients, as well as in refractory DLBCL as a third-line agent.

While mechanistically, XPO1 inhibition is a unique strategy, widespread clinical applicability has been confronted by treatment-emergent adverse events exclusive for the drug class. Though its role in multiple cancer hallmarks is considered a strength, it could be a challenge because it inhibits many essential nucleo-cytoplasmic shuttling processes. Finally, further investigations into prognostic biomarkers are also needed to identify patients with tumors that will be selectively sensitive to XPO1 inhibition. Also, patient and clinician acceptance of TEAEs would likely be higher if there was a significantly enhanced likelihood of clinical response.

## Supplementary information

Supplemental table 1
