# Supplementary material for: Selective inhibition of nuclear export: a promising approach in the shifting treatment paradigms for hematological neoplasms
Source: Leukemia. 2022 Jan 29;36(3):601–12. doi: 10.1038/s41375-021-01483-z (PMC8885406; doi:10.1038/s41375-021-01483-z)
Supplement: Supplementary file 1 — Supplemental table 1 [file 41375_2021_1483_MOESM1_ESM.docx]

Supplementary table 1: Few ongoing clinical trials with SINE compounds in different hematological neoplasms

| Clinical trial identifier/ Phase | Disease | Intervention | Status |
| --- | --- | --- | --- |
| NCT03147885  Phase 1b/2 | Phase 1: N/D indolent and DLBCL as well as R/R indolent B cell lymphomas  Phase 2: | N/D DLBCL | Phase 1 completed. Phase 2 recruitment ongoing (N=44). |
| NCT04640779  Phase 1b | R/R DLBCL or mantle cell lymphoma | Choline Salicylate  and Selinexor | Active not yet recruiting (N=39). |
| NCT02741388  Phase 1b | R/R B-cell malignancies | Group A: Selinexor + R-DHAOx for 3 cycles  Group B: Selinexor + R-GDP for 3 cycles | Recruiting (N=60). |
| NCT04442022  Phase 2 open label/3 double blinded | R/R DLBCL | RGDP±selinexor (S).  Phase 2: two cohorts (40 mg or 60 mg) with R-GDP, for up to 6 cycles, f/b 60 mg selinexor.  Phase 3: SR-GDP vs. standard R-GDP + placebo, for up to 6 cycles, f/b by placebo or 60 mg selinexor | Recruiting (N= 501). |
| NCT02199665  Phase 1 | Relapsed and R/R MM | selinexor, carfilzomib, and dexamethasone | Recruiting (N=100). |
| NCT04661137  Phase 2b, non-randomized (2 arms with additional exploratory arm) | Refractory MM treated with prior any carfilzomib-containing (arm 1), any pomalidomide-containing (arm 2) or any daratumumab-containing regimen (exploratory arm). | Selinexor 60 mg or 80mg in phase 2b part and Selinexor 100 mg in exploratory arm with carfilzomib or pomalidomide and daratumumab respectively. | Recruiting (N=96). |
| NCT02780609  Phase 1/2 | Before autologous SCT for MM | Selinexor in combination with high-dose melphalan | Recruiting (N=46) |
| NCT03589222  Phase 2 | R/R MM | Selinexor (100mg flat dose weekly for 4 weeks) in combination with daratumumab, bortezomib, and dexamethasone | Recruiting (N=62) |
| NCT03955783  Phase 1b | R/R Acute myeloid leukemia, DLBCL and other NHL. | Selinexor and Venetoclax | Recruiting (N=78). |
| NCT03627403  Phase 2 | Myelofibrosis refractory or intolerant to JAK1/2 inhibitors | Selinexor 80 mg once weekly | Recruiting (N=56). |

DLBCL, diffuse large b-cell lymphoma; DHAOx, dexamethasone, ara-c and oxaliplatin; MM, multiple myeloma; N/D, newly diagnosed; R-GDP, rituximab, gemcitabine and cisplatin;

R/R, relapse/refractory; SCT, stem cell transplant.
